# Optimizing Preprocessing and Analysis Pipelines for Single-Subject fMRI: 2. Interactions with ICA, PCA, Task Contrast and Inter-Subject Heterogeneity

**DOI:** 10.1371/journal.pone.0031147

**Published:** 2012-02-27

**Authors:** Nathan W. Churchill, Grigori Yourganov, Anita Oder, Fred Tam, Simon J. Graham, Stephen C. Strother

**Affiliations:** 1 Department of Medical Biophysics, University of Toronto, Toronto, Ontario, Canada; 2 Rotman Research Institute, Baycrest, Toronto, Ontario, Canada; 3 Institute of Medical Sciences, University of Toronto, Toronto, Ontario, Canada; 4 Imaging Research, Sunnybrook Research Institute, Toronto, Ontario, Canada; Institute of Psychology, Chinese Academy of Sciences, China

## Abstract

A variety of preprocessing techniques are available to correct subject-dependant artifacts in fMRI, caused by head motion and physiological noise. Although it has been established that the chosen preprocessing steps (or “pipeline”) may significantly affect fMRI results, it is not well understood how preprocessing choices interact with other parts of the fMRI experimental design. In this study, we examine how two experimental factors interact with preprocessing: between-subject heterogeneity, and strength of task contrast. Two levels of cognitive contrast were examined in an fMRI adaptation of the Trail-Making Test, with data from young, healthy adults. The importance of standard preprocessing with motion correction, physiological noise correction, motion parameter regression and temporal detrending were examined for the two task contrasts. We also tested subspace estimation using Principal Component Analysis (PCA), and Independent Component Analysis (ICA). Results were obtained for Penalized Discriminant Analysis, and model performance quantified with reproducibility (R) and prediction metrics (P). Simulation methods were also used to test for potential biases from individual-subject optimization. Our results demonstrate that (1) individual pipeline optimization is not significantly more biased than fixed preprocessing. In addition, (2) when applying a fixed pipeline across all subjects, the task contrast significantly affects pipeline performance; in particular, the effects of PCA and ICA models vary with contrast, and are not by themselves optimal preprocessing steps. Also, (3) selecting the optimal pipeline for each subject improves within-subject (P,R) and between-subject overlap, with the weaker cognitive contrast being more sensitive to pipeline optimization. These results demonstrate that sensitivity of fMRI results is influenced not only by preprocessing choices, but also by interactions with other experimental design factors. This paper outlines a quantitative procedure to denoise data that would otherwise be discarded due to artifact; this is particularly relevant for weak signal contrasts in single-subject, small-sample and clinical datasets.

## Introduction

Blood Oxygenation Level Dependent fMRI (BOLD fMRI) is an invaluable tool for non-invasive studies of sensory, cognitive and motor neuroscience, and more recently, a range of clinical applications including pre-surgical planning (see review by Fernandez et al. [1]), assessing stroke recovery (reviewed in [2], [3]), and quantifying the effects of therapeutic interventions, e.g. [4]–[6]. However, fMRI is limited by a relatively poor contrast-to-noise ratio (CNR) and strong, structured sources of artifact. The predominant artifact sources are typically subject-specific, and include effects of head motion, respiration and pulsatile blood flow. To reduce artifacts, a variety of denoising algorithms have been developed, ranging from generalized denoising (e.g. subspace selection) to artifact-specific correction (e.g. motion correction).

**Figure 1:**
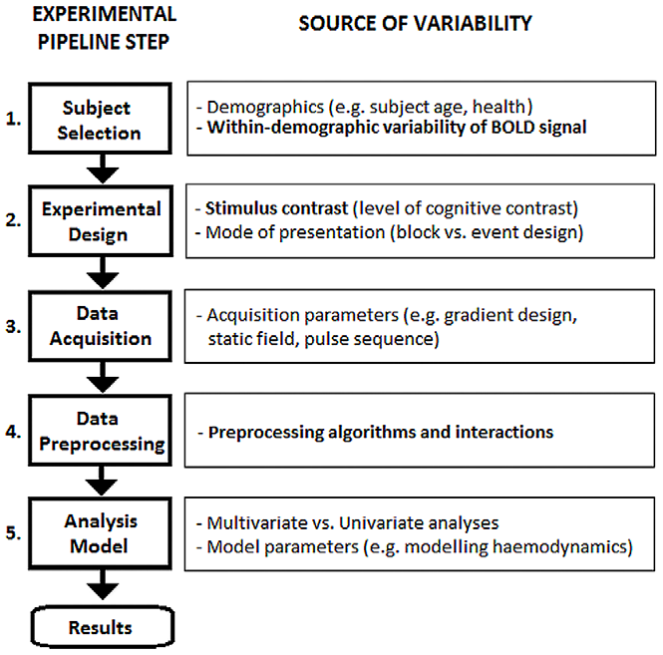
Schematic of the steps in the BOLD fMRI experimental pipeline. An fMRI experiment consists of five major steps; choices at each step significantly influence signal and noise in the results, and may interact with one another. This paper focuses on interactions of individual-subject variability (Step #1), task-contrast effects (Step #2) and preprocessing (Step #4).

In recent years, it has been shown that the chosen set of preprocessing methods (the “pipeline”) significantly impacts the sensitivity and specificity of measured fMRI signals [7]–[17]. It is therefore important to optimize pipeline choices, as better denoising improves signal detection and allows researchers to retain artifact-corrupted data that would otherwise have been discarded from analyses. This is particularly relevant for studies of aging and clinical groups, where signal is weaker, and head motion and physiological noise have a greater impact on fMRI data than for young normal controls [59], [63]. However, there is currently no consensus in the literature on the optimal methods for denoising fMRI data. A potential complication is that preprocessing is not performed in isolation; the full data-analysis pipeline, from experimental design to final results, consists of five discrete steps, displayed in Figure 1. Each of these steps influence signal and noise in fMRI, and thus may interact with preprocessing choices. This paper will focus on the interactions of data preprocessing choices (Step #4) with both between-subject variability (Step #1) and experimental contrast design (Step #2), as there is currently limited information regarding how these steps interact.

Between-subject variability arises in fMRI data, in both the BOLD signal (via neuronal responses related to behaviour and subsequent neurovascular coupling) and artifact sources (including head motion, respiration and heart rate). It is therefore expected that the preprocessing set optimizing signal detection may vary significantly between subjects. For example, motion correction may be optimal for subjects with extensive head motion, and suboptimal for subjects with minimal movement. A number of studies have shown the importance of optimizing the choice of commonly-used preprocessing steps, such as spatial smoothing, temporal detrending and motion correction, on a subject-by-subject basis [8], [16]–[20]. Subspace selection methods are also popular for individually-optimized preprocessing. For example, Principal Component Analysis (PCA) is often used to optimize subspace selection, by denoising and regularizing data prior to analysis [8], [16]–[18]. Independent Component Analysis (ICA) techniques are also popular individually-optimized preprocessing methods, although they are rarely recognized as such; nonetheless, the estimated ICA noise subspace is typically determined on a subject-specific basis. This includes both manual selection of artifact [21]–[24] and quantitative selection methods, employing spatiotemporal priors [25]–[28]. These studies suggest that for fMRI analyses, the standard approach of applying a single set of pipeline steps (a fixed pipeline) to all subjects offers sub-optimal signal detection. However, it is not well understood how subject-specific preprocessing is influenced by other parts of the data-analysis pipeline.

One such source of pipeline variability is the experimental task design. The stimulus type and presentation method determine both measurable brain activation and potential artifacts. The signal/noise trade-offs between block and event-related designs have been previously explored; block designs have greater signal detection power and are less sensitive to haemodynamic response function (HRF) modelling choices [29], whereas event-related designs better estimate BOLD temporal dynamics [30]–[32]. The strength of the task-dependent BOLD contrast may also constitute an important task-design issue. For typical fMRI analyses, experimenters tend to process data with whichever methods are sufficient to extract interpretable brain activation. For strong, spatially localized activations, such as block design visuo-motor tasks, BOLD response may be reliably measured with standard preprocessing, including motion correction with basic physiological noise correction and/or temporal detrending, and univariate analyses (e.g. [33], [34]). By comparison, weaker BOLD signals and functionally connected brain networks are extracted using more extensive preprocessing, including ICA [22], [35], [36] and combinations of global signal normalization, removal of white-matter and CSF timeseries, and motion parameter regression [22], [37]. These findings suggest the hypothesis that specialized pipeline choices are increasingly important for detecting weaker BOLD contrasts temporally varying brain networks. However, to our knowledge, the interaction effects underlying this hypothesis have not been directly examined in fMRI.

Previous studies have focused on standard preprocessing methods and subject heterogeneity [7], [8], [16]–[20]. In particular, this paper is an extension of Churchill et al. [17], which examined interactions of rigid-body motion correction, motion parameter regression, physiological noise correction and temporal detrending. These pipelines were applied to data collected from an fMRI adaptation of a clinical behavioural task, the Trail-Making Test [40], [41], [45], for a strong visuo-motor contrast. The paper compared individual-subject pipeline optimization to an optimal fixed pipeline, demonstrating that individual optimization consistently improves signal detection, as well as the overlap of activation patterns between subjects. The present work extends these findings, as we (a) expanded the tested set of pipelines to evaluate adaptive ICA denoising procedures compared to PCA and other standard preprocessing choices, and (b) examine how optimal pipelines vary as a function of task contrast. These expanded pipelines were applied to the subject data collected from the fMRI-adapted Trail-Making Test, using two levels of contrast for comparison: strong visuo-motor activation, and a second contrast with much weaker changes in distributed cognitive activation networks. The data were analyzed using multivariate Penalized Discriminant Analysis (PDA) on an optimized principal component (PC) basis [39].

Consistent with prior work, we measured the performance of pipeline choices in the data-driven NPAIRS (Nonparametric Prediction, Activation, Influence, and Reproducibility reSampling) framework [38], [39]. For this technique, split-half resampling was used to cross-validate results, based on metrics of spatial reproducibility and temporal prediction accuracy. Reproducibility (*R*) is used to quantify the robustness of statistical parametric maps (SPMs) under resampling. Prediction (*P*) measures how accurately estimated model parameters from a training dataset can predict the experimental condition under which brain scans from an independent set are acquired.

The work is divided into three sections. In the first, we used simulation data to demonstrate that individual-subject pipeline optimization is not a biased procedure, relative to fixed-pipeline optimization. This establishes that individual-subject optimization is not more sensitive to spurious activations (i.e. fitting to noise) than the fixed preprocessing used in the majority of published fMRI studies. For subsequent sections, we examine the interaction of task design, subject heterogeneity and preprocessing in experimental data. In the second section, we demonstrate a procedure for identifying the optimal fixed pipelines for each task contrast, using a combination of (*P*,*R*) metrics and SPM patterns, and we compare results between task contrasts. In the third section, for both contrasts, we show improved signal detection for individual-subject optimization relative to fixed-pipelines, with improved within-subject (*P*,*R*) measures and overlap between individual subject SPMs. The implications of these results are then discussed in detail. For this paper, commonly-used terms and abbreviations are defined in Table 1.

**Table 1:** Commonly used in-text abbreviations and definitions.

PDA	Penalized Discriminant Analysis
PCA	Principal Component Analysis
PCA_full_	First PCA in PDA analysis, performed on full data matrix
PCA_split_	Second PCA in PDA analysis, performed on split-half matrix
(*P*,*R*)	(Prediction, Reproducibility)
MC	Motion Correction
MPR	Motion Parameter Regression
DET	Legendre polynomial DETrending
RET	RETROICOR Physiological Noise Correction
ICA_M_	Independent Component Analysis, MELODIC algorithm
ICA_P_	Independent Component Analysis, PESTICA algorithm
*strong*	Strong cognitive contrast (Task vs. Control)
*weak*	Weak cognitive contrast (Task B vs. Task A)
***FIX***	Optimal fixed preprocessing set, applied to all subjects
***IND***	Individually-optimized preprocessing set
***FIX_PPL_/FIX_PC_***	Fixed pipeline/fixed PC subspace
***FIX_PPL_/IND_PC_***	Fixed pipeline/individually optimized PC subspace
***IND_PPL_/IND_PC_***	Individually-optimized pipeline/individually optimized PC subspace
**ROC**	Receiver Operating Characteristic curve
**TPF/FPF**	True Positive Fraction/False Positive Fraction

## Methods

In this paper, we compare the pipeline set minimizing average distance *D*(*P*,*R*) from perfect prediction and reproducibility D(*P* = 1,*R* = 1) across all subjects (fixed pipelines), to choosing the pipeline that minimizes *D*(*P*,*R*) specific to each subject (individually-optimized pipelines). In the sections below, we describe how this is tested on both simulated (Simulation Methods) and experimental data (Experimental Data and Contrast Design), for strong and weak cognitive contrasts. This is followed by an outline of the tested preprocessing steps (Data Preprocessing) and multivariate analysis model (Analysis Methods). In Measuring Pipeline Performance, we also establish the metrics used to identify optimal pipelines. Then, because fixed preprocessing is the standard methodology for fMRI experiments, we use simulated data to demonstrate that our individual-subject optimization method is not biased, relative to fixed optimization (Testing Simulated-Data Bias of Individual Subject Optimization). In Fixed-Pipeline Optimization of Experimental Data, we outline a procedure for identifying the optimal fixed pipeline in experimental data. We use the fixed-pipeline results to (1) determine the optimal combination of ICA and PCA for estimating the signal subspace (Optimizing Subspace Selection for Experimental Data), and (2) compare fixed preprocessing against the individually-optimized pipeline choices described in Individual-Subject Optimization of Experimental Data.

### Simulation Methods and Data

**Figure 2:**
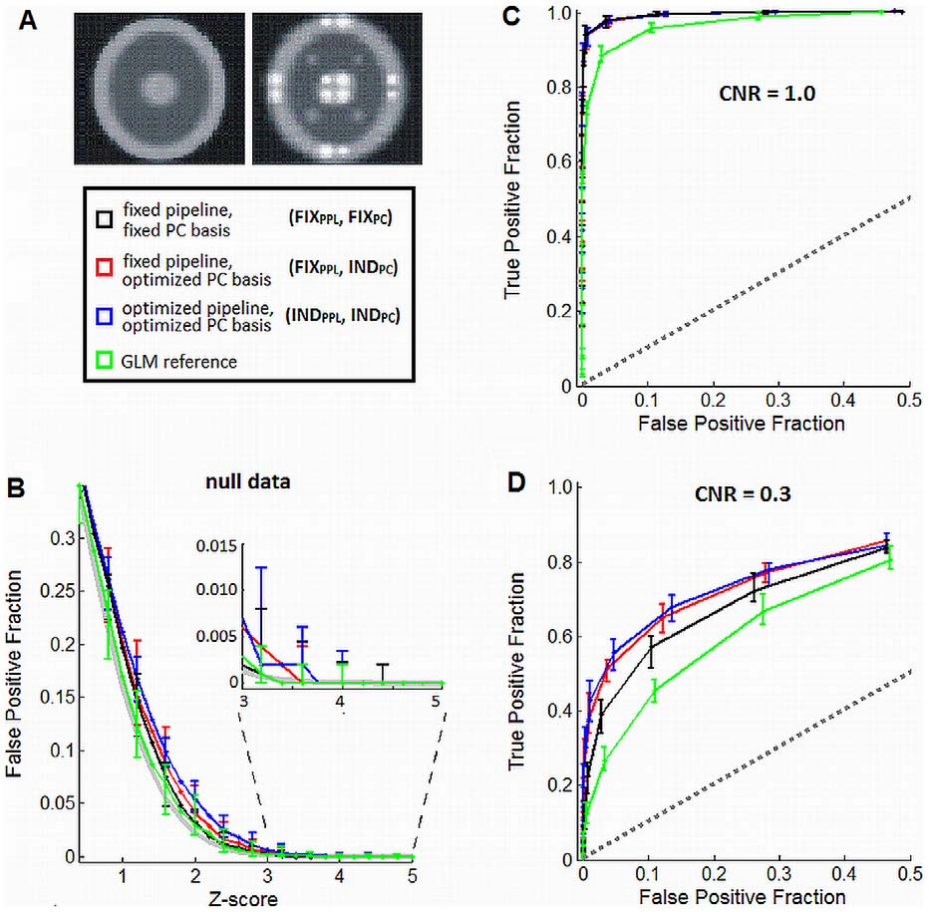
Simulation results, testing for bias in pipeline optimization. (A) simulated phantom during baseline (left) and activation (right). (B) median False-Positive Fraction (FPF) as a function of Z-score threshold in null data, with error bars showing the range of FPF across activation loci. The expected null distribution (Normal curve of zero mean, unit variance) is plotted as a gray line. (C–D) ROC curves plotting median TPF vs. FPF over the 16 signal loci (error bars show the range of TPF). Results are shown for 3 PDA pipelines, and a General Linear Model (GLM) reference; the line of no signal detection TPF = FPF is also plotted (dashed line). Fixed pipelines have no preprocessing, and the fixed PC basis is the median basis minimizing distance *D* from (*P* = 1,*R* = 1) across all samples (PC dim. = 1 and 3 for CNR = 1.0 and 0.3). The optimized pipeline and PC basis are chosen to minimize *D*, separately for each sample.

The simulation analyses provide a conservative test of optimization bias: for this paper, we generated samples from a fixed Gaussian distribution, treating samples as repeated observations of a single “subject” for whom we optimize pipelines. This is the case in which individual-sample optimization is least necessary, providing a “null” dataset for optimization. Our hypothesis is that even individual-sample optimization of this dataset does not bias results by fitting to noise, which would produce spurious activations. The simulation methods and parameters are briefly outlined; for full details on the model, see [42], [43]. The synthetic data simulated a brain slice during a block-design experiment, with 10 “activation” images, followed by 10 “baseline” images (in total, 200 scans per sample). We used a single-slice brain model, with simulated grey and white matter (GM and WM); Fig. 2(A) shows baseline (left) and activation (right) images. The model included simulated Gaussian noise and signal (during activation scans), spatially smoothed and temporally convolved with the canonical HRF described in [44]. Functional connectivity of a distributed brain network was simulated with 16 activation loci (4 in WM, 12 in GM); the expected correlation of time series across simulated activation loci was ρ = 0.5. We generated a set of 500 simulated runs from the multivariate Gaussian distributions, for Contrast-to-Noise ratios (CNRs) of CNR = 1.0 and CNR = 0.3, in order to compare nominally “strong” and “weak” cognitive contrasts; in addition, we generated a “null” dataset, with no activation loci present.

### Experimental Data and Contrast Design

BOLD fMRI data were acquired on a 3.0 T MR scanner (MAGNETOM Tim Trio, VB15A software; Siemens AG, Erlangen, Germany) with a 12-channel head coil. A T1-contrast anatomical scan was obtained (oblique-axial 3D MPRAGE, 2.63/2000/1100 ms TE/TR/TI, 9° FA, 256×192×160 matrix size, 1×1×1 mm voxels), followed by BOLD fMRI data (2D GE-EPI, 30/2000 ms TE/TR, 70° FA, 64×64×30 matrix size, 3.125×3.125×5 mm voxels). Subjects received a 15 minute orientation session in an MRI simulator, and then performed 2 task runs in the scanner, separated by approximately 10 minutes of other neurobehavioural tests. To minimize non-stationary learning effects, observed in subjects' behavioural performance for the first run, only the scan data of run 2 were analyzed for this study.

The last two sections of Methods use experimental data derived from a standard behavioural assessment task, the Trail-Making Test [40], [45], which was adapted for the fMRI environment. The task consisted of 2 types of task stimuli: *TaskA*, in which numbers 1–14 were displayed in pseudo-random locations on a viewing screen, and *TaskB*, in which numbers 1–7 and letters A-G were displayed. Subjects drew a line connecting items in sequence (1-2-3-4-…) or (1-A-2-B-…), connecting as many as possible over a 20 s block duration, while maintaining accuracy. A *Control* stimulus was presented after each task block, in which subjects traced a line from the center of the screen to a circle (randomly placed at a fixed radius from the center of the screen) and back over 2 s, repeated 10 times. Subjects performed a 4-block, 40-scan, Task+Control epoch of *TaskA-Control*-*TaskB*-*Control* twice per run, with 2 runs per subject. Tracing was performed with an MRI-compatible writing tablet and stylus [41], with subjects monitoring their performance on a projection screen. Data were acquired from 24 young, healthy volunteers, aged 20–33 yrs with median age 24 yrs (14 female). Subjects were confirmed right-handed via the Edinburgh Handedness Inventory [46], and screened for cognitive and neurological deficits, by self-report and Mini-Mental Status Examination [47], with median score 30 and range 28 to 30 (out of 30). All participants gave written informed consent for their participation and the experiment was conducted in the Rotman Research Institute, Baycrest Hospital, with the approval of the Baycrest Research Ethics Board.

Two levels of experimental contrast were examined. The first contrast was the comparison of *Task* (*TaskA*+*TaskB*) vs. *Control* condition scans, denoted the “*stronger*” contrast due to comparison of high-level visuo-spatial search tasks against the simpler baseline tracking task. Activations consistent with both task and Default-Mode Network (DMN) have been previously found in a smaller group for *strong* contrast [17], primarily in regions associated with visual and motor recruitment. The second contrast compared *TaskB* vs. *TaskA* conditions, subsequently referred to as the “*weaker*” contrast. This contrast is thought to reflect primarily cognitive components, specifically increased recruitment of attention, set-switching and working memory domains [40], [48]. Preliminary testing confirmed that *strong*-contrast analyses showed consistently higher Z-scores in regions of maximum activation, and higher mean (*P*, *R*) values, compared to the *weaker TaskB* vs. *TaskA* (see RESULTS: Individual-Subject Optimization of Experimental Data).

### Data Preprocessing

Experimental data were preprocessed with AFNI utilities [49] in the following order. Rigid-body motion correction (MC) was applied via *3dvolreg*, registering all volumes to the 40^th^ volume within a run, using a weighted least-squares cost function and Fourier interpolation. The “twopass” setting was applied, which performs coarse initial registration at lower resolution, then registration in the native voxel resolution. Images then had slice-timing correction with Fourier interpolation (via *3dTshift*), and spatial smoothing with a 6.0 mm FWHM Gaussian kernel (with *3dmerge*); these two steps were held fixed for all pipelines. Physiological noise correction was performed using RETROICOR (RET) to regress out signal correlated with cardiac and respiratory phases [50], measured with a finger photoplethysmograph and respiratory belt, respectively. Temporal detrending was performed using a Legendre polynomial basis set ranging from zeroth to fifth order (DET0–5). We also regressed out head motion artifacts based on the subject motion parameter estimates (MPEs) obtained from MC. For motion parameter regression (MPR), we performed PCA on the six MPE time-courses, and used the two largest-variance principal components as regressors [51]. For all subjects, these two components accounted for more than 85% of estimated temporal motion variance, which allowed us to maximize the amount of head motion variance accounted for, while minimizing loss of power and collinearity effects due to unnecessary parameterization. Detrending and motion parameter regression were performed concurrently via multiple linear regression.

We also integrated two ICA-based denoising methods into the preprocessing pipeline, performed after spatial smoothing, applying at most one ICA method per pipeline. This allowed us to test whether ICA is an important denoising step in the optimized pipeline, before performing PDA on a regularized PCA subspace (see Analysis Methods below), and whether the choice of ICA technique has a significant impact. We selected two methods that are widely used, described as follows:

1. PESTICA (ICA_P_): a data-driven estimator of cardiac and respiratory effects that uses the Infomax algorithm with enforced temporal independence [28]. PESTICA identifies 4 cardiac and 2 respiratory time-series regressors, based on spatial weighting maps of cardiac and respiratory effect, and manual selection of the artifact's temporal power-spectrum band (using code available at www.nitrc.org/plugins/mwiki/index.php/pestica:MainPage). For each subject dataset, we manually estimated the spectral peak range nearest to the suggested maxima of 17 bpm and 60 bpm (respiratory and cardiac peaks, respectively).
2. MELODIC manual selection (ICA_M_): we also employed FSL's MELODIC package (www.fmrib.ox.ac.uk/fsl/melodic2/index.html) to estimate and remove artifacts, based on visual inspection of Independent Components (ICs). This model performs Probabilistic PCA (a probabilistic Gaussian model, with components obtained via Expectation-Maximization) to estimate the signal/artifact source subspace, followed by a non-orthogonal rotation of component vectors to maximize spatial independence via negentropy measures [52]. We performed a conservative selection of the resultant noise ICs, based on spatial and temporal characteristics consistent with motion and physiological artifact. We discarded only those components in which artifact was predominant; see supplementary Text S1 for a summary of the component selection criteria.

These two methods were selected, as they allowed us to compare the impact of adding: (1) a model driven by known physiological noise priors and with fixed dimensionality (ICA_P_) and (2) an unconstrained source selection method with variable dimensionality (ICA_M_) relative to (3) PDA using regularized PCA components without any ICA.

We obtained EPI data preprocessed with all possible combinations of the data preprocessing steps MC, RET, MPR, ICA_P_/ICA_M_ and DET0–5 included/excluded from the pipeline. This generated 2^3^×3×6 = 144 preprocessed datasets for each subject, all of which were then analyzed. For the simulation datasets, the *3dvolreg* and ICA could not be performed (due to the 2D geometry and Gaussian signal structure, respectively). We applied a restricted preprocessing set including DET0–5, RET and MPR. We randomly selected cardiac, respiratory and motion measurements from one of the 24 subjects to provide a basis for regression, which was then fitted to the simulated datasets, to perform the latter two preprocessing choices. This produced 2^2^×6 = 24 different pipelines in the simulated data.

### Analysis Methods

The Penalized Discriminant Analysis (PDA) was performed by applying two PC decompositions on preprocessed data from run 2; first on the full data matrix composed of both task epochs (PCA_full_), then individually on each task epoch (or split-half) of the run 2 data (PCA_split_). This was followed by Linear Discriminant Analysis (LDA) on the PCA_split_ subspace, to obtain a classifier brain map, or SPM, for each task epoch; full analysis model details are given in [8], [39]. For PCA_full_, we kept the 35% of PCs accounting for most variance, which optimized average distance *D*(*P*,*R*), across all subjects and the two task contrasts. The subspace for each split-half matrix (PCA_split_) was then selected using a step-up process, starting with the first two PCs accounting for the most variance, and sequentially adding the PC that accounted for the most remaining variance; the optimal basis size was chosen as the sequential set of PCs that minimized *D*(*P*,*R*) across splits. The SPMs of the optimized PCA_split_ basis were used to calculate (*P*,*R*) metrics and rSPM(Z) (defined below). In total, (144 preprocessing sets)×(2 task contrasts) = 288 sets of analysis results were obtained per subject. For simulations, we obtained 24 sets of analyses per run, for both CNRs.

### Measuring Pipeline Performance

Given that there is no measure of “ground truth” in fMRI experiments by which to compare different pipeline results, we employed data-driven metrics of prediction accuracy and reproducibility (*P*,*R*) in the NPAIRS split-half resampling framework, and identified the pipeline choices optimizing these measures. This method, developed in Strother et al. [38] and extended to fMRI by LaConte et al. [8], is briefly outlined; details of the metrics and reproducible SPM estimation are provided in supplementary Text S2 and Figure S1. For a given subject's dataset, scans were split into two pseudo-independent groups (i.e., 1^st^ and 2^nd^ halves/epochs of a single run) and analysis performed separately on each split-half, producing two statistical parametric maps (SPMs) of brain activations. The *R* metric was estimated between splits by Pearson correlation of the split-half SPMs' voxel values. We also generated a reproducible SPM, with a Z-transformed value at each voxel (rSPM(Z)); this is obtained from the linear combination of the 2 split-half SPMs maximizing their reproducible signal [38]. Prediction *P* was computed using analysis results of split 1 to predict the class, or brain state, of individual scans in split 2 and vice-versa, via Bayes' posterior probability. Accuracy of predictions was averaged across splits. The posterior probability for the 2-class PDA model, which is equivalent to LDA on an optimized PCA basis, is estimated from the multivariate Gaussian distribution [39], [61].

Both *R* and *P* may range from 0 to 1, with perfect performance at 1; *P* = 0.5 corresponds to random guessing for 2-class analyses. Both metrics measure equally important neuroscientific targets (i.e. a model that (i) generates a robust activation map and (ii) accurately predicts brain-state), but also capture important tradeoffs in model parameterization; maximizing *P* typically comes at the expense of *R* and vice-versa (see [62] and details in Text S2). In order to jointly optimize both metrics, model performance was defined as Euclidean distance *D* from perfect prediction and reproducibility (*P* = 1,*R* = 1). Better pipeline performance is given by smaller *D*, with *D* = 0 indicating perfect model performance.

### Testing Simulation-Data Bias of Individual Subject Optimization

We began by testing for bias in individual-subject optimization, compared to the more commonly-used fixed pipeline optimization. Our goal is thus to compare *relative* bias between fixed/individually optimized pipelines, for a simulated mixture of multivariate network signal and noise, which has direct implications for experimental fMRI data. For each of the 16 central pixels of the activation loci, we also randomly selected a non-active pixel (for the same background signal level), and counted true positive/false positive fractions over the 500 samples (TPF/FPF), for a range of Z-score thresholds; this produced Receiver Operating Characteristic (ROC) curves for each signal locus. We computed median ROC curves and ranges of TPF/FPF, over all 16 signal loci (the latter shown as error bars), for CNR = 0.3 and 1.0.

Given the lack of structured noise or motion in these data, only the PC subspace selection during PDA analysis was expected to provide strong control of the Gaussian noise; hence, we examined individual-sample optimization of both the regression models (DET, MPR and RET) and PC basis. The TPF and FPF were compared for 3 pipelines: fixed preprocessing with fixed PC dimensionality for PDA (*FIX_PPL_+FIX_PC_*); fixed preprocessing with individually-optimized PC dimensionality (*FIX_PPL_+IND_PC_*); and individually-optimized preprocessing with individually-optimized PC dimensionality (*IND_PPL_+IND_PC_*). The optimal fixed PC dimension was selected as the median of all PC dimensions that minimized *D*(*P*,*R*) for each sample; the optimal fixed pipeline was identified using the ranking procedure described below in METHODS: Fixed-Pipeline Optimization of Experimental Data. Individually-optimized pipelines were the preprocessing steps and PC dimensionalities that minimized *D*(*P*,*R*), specific to each sample (equivalent to METHODS: Individual-Subject Optimization of Experimental Data).

We compared the median ROC curves of the three pipelines for CNR = 0.3 and 1.0, and also plotted FPF as a function of Z-score threshold for the rSPM(Z)s of the null data, as we were concerned with individual-sample optimization measuring spurious activations in the absence of signal. We also plotted ROC curves for a General Linear Model (GLM) analysis, with Ordinary Least Squares estimation and a binary task-design structure, to provide a univariate reference curve. We directly tested whether the method of pipeline optimization affects signal detection, by computing the area under each sample ROC curve for FPF<0.1 for all three pipeline sets, as we are interested in signal detection for this low FPF range. We tested for significant changes between pipelines in the 16 partial ROC area measurements, using nonparametric paired-sample Wilcoxon tests.

### Fixed-Pipeline Optimization of Experimental Data

We directly examined preprocessing effects, by comparing performance of the 144 fixed pipeline combinations of RET, MC, MPR, ICA and DET0–5, for each task contrast. A fixed-pipeline analysis procedure was established, to (a) identify optimal pipelines based on (P,R) metrics, then (b) characterize the spatial structure of the pipelines' rSPM(Z)s using the DISTATIS clustering technique [53], [54]. This procedure identifies the optimal fixed pipeline, in order to provide a fair comparison for individual-subject optimization. We briefly describe these methods as follows; Supplementary Text S3 and Figure S2 provide extensive details on the fixed-pipeline optimization procedures.

1. Optimizing (P,R) metrics: we used a non-parametric procedure to test for a significant ordering in pipeline performance that is common across all subjects (first applied in [17]). For each subject, pipelines are ranked by their *D*-metric; provided there is a significant pipeline ranking, we identify the set of *L* optimal, highest-ranked pipelines, based on an *α* = 0.05 critical-difference bound. The set L was estimated for each task contrast.
2. Characterizing SPM spatial structure: pipelines with similar (*P*, *R*) may be driven by different spatial patterns [17]. Therefore, for the set of *L* fixed pipelines which have statistically indistinguishable (*P*, *R*) distributions, we tested for significant differences in rSPM(Z) patterns. We performed the three-way multidimensional scaling of DISTATIS [53], [54], which provides a PC-space representation of the similarity between pipeline rSPM(Z)s most common across all subjects. The novel features of this technique are that (i) we obtain a denoised estimate of the most common rSPM(Z) similarity pattern across all subjects, and (ii) we perform Bootstrap resampling on this similarity pattern, to produce empirical, nonparametric 95% confidence ellipses for each pipeline. The *L* pipelines were examined for clustering, defined as overlap in DISTATIS 95% confidence ellipses, indicating that their rSPM(Z)s are not significantly distinguishable.
3. Maximizing overlap between subject SPMs: the DISTATIS method allows us to select an optimal pipeline based on SPM characteristics; however we are also interested in maximizing fixed-pipeline performance metrics. Therefore, we chose the optimal fixed pipeline from the *L* candidates (denoted *FIX*) as the one with greatest average Jaccard overlap between subject rSPM(Z)s (the intersection/union of significantly active voxels, for a False-Discovery Rate threshold (FDR) = 0.05). This pipeline is both optimal in (*P*,*R*), and maximizes the consistency of activation loci between subjects.

The 95% confidence ellipses were plotted for the *L* optimal pipelines of each task contrast. We also plotted a mean rSPM(Z) from each DISTATIS 95% confidence cluster, using a single representative pipeline, to demonstrate that the clustered pipeline groups generate significantly different spatial structures. Finally, we identified the optimal FIX pipeline for each contrast.

### Optimizing Subspace Selection for Experimental Data

We also tested whether ICA may be used to effectively estimate the fMRI signal subspace, compared to PCA. This is a separate issue from whether ICA effectively removes artifact, and has implications for the optimal method of signal estimation in multivariate fMRI analyses. The use of ICA to estimate functional brain networks has become of increasing interest in recent years; however it is not clear whether it provides better estimation of the fMRI signal than PCA-based predictive analysis models. We therefore compared 4 different subspace selection methods, for the optimal fixed preprocessing pipeline of each task contrast (obtained using the procedure outlined in the previous section).

For this section, we compared different subspace selection methods to a “baseline” reference of data without dimensionality reduction; for each subject, we obtained this baseline by performing the 2 PCAs prior to PDA analysis, without discarding any components (i.e. no subspace selection), and obtained the (*P*,*R*) values. We then measured (*P*,*R*) of analysis results for (ICA): an optimized ICA subspace, estimated from MELODIC, with artifact components discarded as outlined in METHODS: Data Preprocessing; (PCA_split_): an optimized PCA subspace based on *D*(*P*,*R*) metric; and (ICA+PCA_split_): ICA denoising, followed by PCA optimization; for these 3 methods, we performed analyses without the initial 35% data reduction in PCA_full_ (see METHODS: Analysis Methods), so that we could directly compare PCA results to denoising with ICA along (performed on the split-halves). We also demonstrated (PCA_full_+PCA_split_) results, so we could compare these subspace selection models to the method used in the rest of the paper. For (ICA), we performed ICA denoising, then transformed the denoised data using PCA prior to PDA, without discarding any components; this is equivalent to a weighted linear combination of the IC components. We then compared the (*P*,*R*) values of the 3 different methods to the “baseline”.

### Individual-Subject Optimization of Experimental Data

The preprocessing combination that minimized *D* for each subject was also determined, for each combination of task-contrast and analysis model, providing the “individually-optimized pipeline set” (*IND*).

For *IND* optimization on experimental data, we require an added step to account for task-coupled motion (TCM), which generates artifact that is task-correlated and reproducible, and thus not controlled by optimizing *D*(*P*,*R*). We used a quantitative procedure to reject pipelines corrupted with motion artifact, when selecting the optimal pipeline for each subject; see Text S4 for details. The effect of *IND* optimization on (*P*,*R*) was examined, relative to the optimal fixed pipeline *FIX*, for both task contrasts. We also examined trends in individually-optimal pipelines, by examining the number of subjects requiring each preprocessing step for optimal model *D*(*P*,*R*), for both task contrasts.

The spatial structure of SPMs for *FIX* and *IND* pipelines was also compared. The *IND* pipelines have been previously found to increase activation overlap between subjects, relative to fixed pipelines [16]–[18]. We hypothesized that *IND* also consistently increases activation overlap between subjects relative to *FIX* for both task contrasts, even though we have explicitly selected *FIX* to maximize activation overlap. An increase in between-subject activation overlap indicates that we are optimizing the detection of functional activations that are consistent across all subjects. This provides independent validation of our optimization process since, unlike *FIX* pipelines, *IND* subject pipelines are optimized independent of one another.

We computed (1) whole-brain reproducibility between all subject pairs, based on the Pearson correlation coefficient, and (2) the overlap of activated brain regions, by thresholding individual subject SPMs at FDR = 0.05 and measuring pairwise Jaccard overlap between all subjects. We measured the mean correlation/overlap of each subject with all others, for both *IND* and *FIX*. Brain regions that show significant change between *IND* and *FIX* were also plotted, estimated via Bootstrap resampling on the mean difference in Z-scores, to find regions with consistent positive/negative change. We plotted mean Z-score change (*IND*−*FIX*) at significant regions, corrected for multiple comparisons at FDR = 0.05. These analyses were performed for both *strong* and *weak* task contrasts.

## Results

### Testing Simulated-Data bias of individual subject optimization

For the fixed pipelines *FIX_PPL_*+*FIX_PC_*, the optimal PC dimensionalities of CNR = 1.0 and 0.3 were 1 and 3 PCs, respectively. The null dataset and both CNRs exhibited significant fixed-pipeline rankings (all *p*<0.01, Friedman test). For all datasets, the DET0 pipeline with “no preprocessing” had the optimal individual ranking and was also chosen as the fixed optimum pipeline. This was expected given the absence of structured noise in the simulations. For individually-optimized datasets of *IND*_PPL_+*IND*_PC_, regression-based preprocessing optimized (*P*,*R*) for a subset of datasets, although the regressors are not designed to control Gaussian noise. For example, at CNR = 1.0, 218/500 samples included RET for optimization and 150/500 included MPR. In addition, samples were optimized with median detrending order 2 and range 0–5, for both CNRs.

Fig. 2(B) plots the median and range of FPF across all 16 loci in null data, as a function of Z-score threshold for the three pipelines and GLM analysis. The expected null distribution is also plotted based on a normal curve of zero mean, and unit variance (gray line). Although *FIX_PC_+FIX_PPL_* had the lowest median FPF, no consistent difference was observed between the pipelines, as all range error bars overlap. In addition, as the Z-score threshold is increased beyond 3.7 (inset on Fig. 2(B); *p*<10^−4^, uncorrected), all pipelines attain zero median FPF. Therefore, for homogeneous Gaussian signal and noise, individual pipeline optimization showed a weak but non-significant increase in FPF compared to fixed pipelines.

Fig. 2(C–D) plot median ROC curves of the three pipelines. For both CNRs, the range error bars overlapped for all non-GLM pipeline ROC curves, although the medians are consistently higher for pipelines with *IND_PC_* in CNR = 0.3. For CNR = 1.0, all pipelines have mean partial ROC area 0.093±0.004, with no significant change for either *IND_PC_+FIX_PPL_* or *FIX_PC_+FIX_PPL_*, relative to *FIX_PC_+FIX_PPL_* (*p* = 0.519 and *p* = 0.850 respectively; paired Wilcoxon test). However, for CNR = 0.3, we measured mean partial ROC areas of 0.034±0.003 (*FIX_PC_+FIX_PPL_*), 0.042±0.005 (*IND_PC_+FIX_PPL_*) and 0.043±0.004 (*IND_PC_+IND_PPL_*). Both *IND_PC_+FIX_PPL_* and *IND_PC_+IND_PPL_* show significantly increased partial ROC area relative to *FIX_PC_+FIX_PPL_* (*p*<0.01 for both), while *IND_PC_+FIX_PPL_* and *IND_PC_+IND_PPL_* are not significantly different (*p* = 0.791). Individual-sample optimization therefore produced either comparable (CNR = 1.0) or improved (CNR = 0.3) signal detection in simulation data, relative to fixed preprocessing.

### Fixed-Pipeline Optimization of Experimental Data

A significant, consistent pipeline *D*(*P*, *R*) ranking was observed for both task contrasts (*p*<0.01, Friedman test), allowing us to identify a subset of optimal pipelines for each contrast (see Text S3). For *strong* contrast, we identified 15 of the 144 pipelines as optimal (Nemenyi test, α = 0.05), listed in Table 2; note that spatial smoothing and slice-timing correction are the only fixed preprocessing steps for all pipelines. Of these fixed pipelines, 14/15 included ICA_M_ and 9/15 included MC, being the most consistently important fixed pipeline steps. No optimal fixed pipeline included ICA_P_ or MPR, indicating that they are suboptimal procedures for this contrast, but ICA_M_ followed by PCA dimensionality reduction is almost uniformly optimal for a fixed pipeline.

**Table 2:** Optimal fixed pipelines for Strong-Contrast data.

Cluster Group	ICA	MC	MPR	RET	DET
**1**		X			2
**2**	M				0
**2**	M				1
**2**	M				2
**2**	M				4
**2**	M				5
**3**	M	X			0
**3**	M	X			1
**3**	M	X			2
**3**	M	X			3
**3**	M	X			4
**3**	M	X		X	0
**3**	M	X		X	1
**3**	M	X		X	2
**3**	M	X		X	4

**Figure 3:**
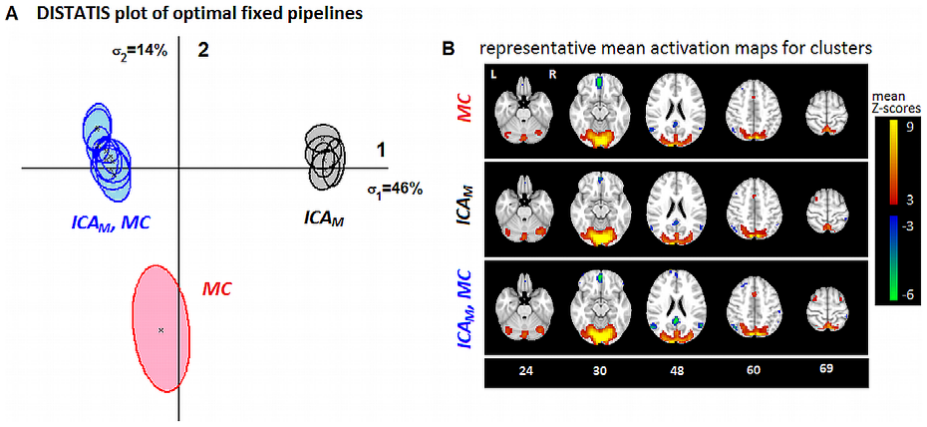
Testing fixed-pipeline spatial structure for *strong* task contrast (Task vs. Control). (A) DISTATIS-space plot, showing the similarity in spatial structure of optimal fixed pipeline SPMs; pipelines with overlapped 95% confidence ellipses have non-significantly different SPMs in this space. Cluster numbering is consistent with Table 2, and labelled with preprocessing steps that are consistent across all pipelines in the cluster, abbreviated: ICA with the MELODIC algorithm (**ICA_M_**), motion correction (**MC**). See Table 2 for the corresponding list of pipelines. (B) mean activation maps, from a representative pipeline of each cluster: {MC,DET2}, {ICA_M_,DET2} and {ICA_M_, MC,DET2} (top to bottom).

The optimal fixed pipeline combinations for *strong* contrast (Task vs. Control), identified via Nemenyi test (*p* = 0.05). Preprocessing includes ICA (M = MELODIC; **P** = PESTICA), motion correction (**MC**), motion parameter regression (**MPR**), physiological noise correction via RETROICOR (**RET**) and polynomial detrending for orders 0–5 (**DET**). Pipelines with the same Cluster Group number have no significant difference in SPMs, based on overlapped 95% confidence ellipses in DISTATIS space, shown in Fig. 3.

Figure 3(A) shows the DISTATIS clustering analysis for fixed-pipeline rSPM(Z)s of the *strong* contrast, where the two plotted dimensions account for 60% of total rSPM(Z) variance. We observe 3 significantly different clusters with non-overlapped 95% confidence ellipses; clusters are labelled with preprocessing step(s) that are consistent for all overlapping pipelines. Fig. 3(B) shows mean rSPM(Z)s from a representative of each cluster, demonstrating that the pipeline groups tend to extract different brain patterns. At a fixed threshold, {ICA_M_,DET2; middle row} exhibits weaker mean task-negative Z-scores, whereas {ICA_M_,MC,DET2; bottom row} shows distinct frontal task-positive activations (slice 69), and {MC,DET2; top row} shows greater signal in the ventral anterior cingulate cortex (vACC) (slice 30). The three optimal fixed pipelines have a significant ranking for between-subject activation overlap (at FDR = 0.05), of {ICA_M_,MC,DET2}>{ICA_M_,DET2}>{MC,DET2} (*p*<0.01, Friedman test); we thus selected {ICA_M_,MC,DET2} as the optimal fixed pipeline *FIX* for *strong* contrast.

We identified 11/144 as optimal fixed pipelines for *weak* contrast based on *D*(*P*,*R*) ranking and permutation testing (α = 0.05), listed in Table 3. The preprocessing optima were more varied, with ICA_P_ now required for 5/11 pipelines, RET required for 6/11 pipelines, and ICA_M_ being part of 4/11 pipelines; the latter is thus less consistently optimal for the *weaker* contrast. In addition, MC was markedly less important, only being part of 2/11 optimal fixed pipelines. In addition, optimal DET order was more consistent, as DET0 or 4 was required to optimize 10/11 pipelines.

**Table 3:** Optimal fixed pipelines for Weak-Contrast data.

Cluster Group	ICA	MC	MPR	RET	DET
**1**	P			X	4
**1**				X	4
**2**				X	2
**3**	P			X	0
**3**	P				0
**3**	P	X			0
**3**	P	X		X	0
**4**	M				0
**5**	M				4
**5**	M			X	4
**5**	M		X		4

**Figure 4:**
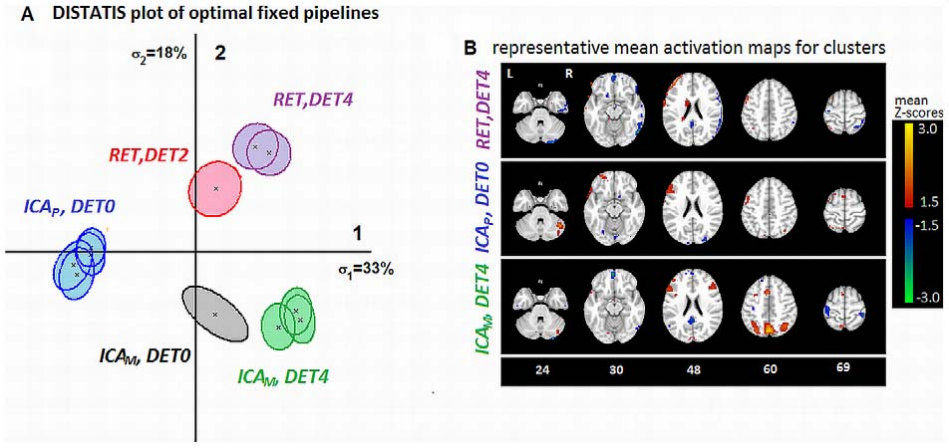
Testing fixed-pipeline spatial structure for *weak* task contrast (TaskB vs. TaskA). (A) DISTATIS-space plot, showing the similarity in spatial structure of optimal fixed pipelines SPMs; pipelines with overlapped 95% confidence ellipses have non-significantly different SPMs in this space. Cluster numbering is consistent with Table 3, and labelled with preprocessing steps that are consistent across all pipelines in the cluster, abbreviated: ICA by MELODIC algorithm (**ICA_M_**) and PESTICA (**ICA_P_**), Physiological Noise Correction with RETROICOR (**RET**) and polynomial temporal detrending (**DET**(order)). See Table 3 for corresponding list of pipelines. (B) mean activation maps, from a representative pipeline of each major cluster: {RET, DET4}, {ICA_P_,DET0}, {ICA_M_,DET4} (top to bottom).

The optimal fixed pipeline combinations for *weak* contrast (TaskB vs. TaskA), identified via Nemenyi test (*p* = 0.05). Preprocessing includes ICA (**M** MELODIC; **P** = PESTICA), motion correction (**MC**), motion parameter regression (**MPR**), physiological noise correction via RETROICOR (**RET**) and polynomial detrending for orders 0–5 (**DET**). Pipelines with the same Cluster Group number have no significant difference in SPMs, based on overlapped 95% confidence ellipses in DISTATIS space, shown in Fig. 4.

Figure 4(A) shows the DISTATIS clustering analysis for fixed-pipeline rSPM(Z)s of the *weak* contrast; the plotted dimensions account for 51% of total rSPM(Z) variance. Compared to *strong* contrast's 3 pipeline clusters, we observed an increase to 5 distinct clusters (see Table 3); the clusters are again labelled with preprocessing step(s) that are consistent for all pipelines within the group. Fig. 4(B) compares mean SPMs from representative pipelines of the 3 largest clusters, as the other 2 are intermediate between these groups. These maps reinforce the importance of spatial testing, as the {RET,DET4} group appears to be corrupted with task-coupled motion, based on substantial apparent activation rimming the brain. The 3 fixed pipelines also exhibited significant ranking for between-subject activation overlap (at FDR = 0.05), of {ICA_M_,DET4}>{ICA_P_, DET0}>{RET,DET4} (*p*<0.06, Friedman test); the {ICA_M_,DET4} pipeline was selected as the optimal fixed pipeline *FIX* for *weaker* contrast.

### Optimizing Subspace Selection for Experimental Data

Tables 4 and 5 show the results of using different combinations of ICA and PCA to optimize data dimensionality for analysis; we measured changes relative to data without any dimensionality reduction. The PCA and ICA-PCA pipelines always significantly improved *D*(*P*,*R*), while ICA alone as estimated in MELODIC was not always significantly beneficial. Performing ICA optimization only significantly improved the *P* metric, for *weak* contrast. By comparison, PCA and ICA-PCA methods improved all metrics for *weak* contrast, and *R* and *D* for *strong* contrast (at the expense of decreased *P*). Therefore, MELODIC ICA is a sub-optimal estimator of the signal subspace for PDA analysis of both task contrasts, requiring further PCA dimensionality reduction. In addition, the 2-step PCA procedure (PCA_full_+PCA_split_), used for analyses in the rest of this paper, generally demonstrates improved median *R*, *P* and *D* relative to the other methods, indicating that it is the better subspace selection method.

**Table 4:** Comparing performance of different subspace estimation methods, for Strong-Contrast data.

	Δ*R*	signif.	Δ*P*	signif.	Δ*D*	signif.
***ICA***	−0.04 [−0.27, 0.11]	<0.01	0.00 [−0.17, 0.15]	0.38	0.04 [−0.11, 0.27]	0.01
***PCA_split_***	0.31 [ 0.04, 0.43]	<0.01*	−0.06 [−0.30, 0.06]	<0.01	−0.29 [−0.40, −0.04]	<0.01*
***ICA*** **+** ***PCA_split_***	0.34 [ 0.24, 0.47]	<0.01*	−0.04 [−0.17, 0.05]	<0.01	−0.33 [−0.47, −0.17]	<0.01*
***PCA_full_*** **+** ***PCA_split_***	0.35 [ 0.19, 0.47]	<0.01*	−0.05 [−0.12, 0.03]	<0.01	−0.34 [−0.48 −0.18]	<0.01*

For *strong* contrast (Task vs. Control), model performance is shown for different subspace estimation methods, relative to full-dimensionality data (i.e. retaining all PCs). The median, [minimum, maximum] changes are shown for prediction (**Δ** *P*), reproducibility (**Δ** *R*) and distance **Δ** *D* from (*P* = 1,*R* = 1), over all single-subject results. Significance is given by Wilcoxon tests, with * indicating significant improvement. We show results for combinations of **ICA** = MELODIC subspace estimation, **PCA_split_** = optimized PC subspace on each data split-half, and **PCA_full_** = retaining 35% of PCs from the full data matrix. Note that (**PCA_full_**+**PCA_split_**) is the subspace selection method used for the rest of the manuscript. Results are shown for optimal fixed preprocessing: motion correction and 2^nd^-order detrending.

**Table 5:** Comparing performance of different subspace estimation methods, for Weak-Contrast data.

	Δ*R*	signif.	Δ*P*	signif.	Δ*D*	signif.
***ICA***	−0.02 [−0.24, 0.47]	0.83	0.19 [−0.36, 0.53]	0.02*	−0.07 [−0.67, 0.29]	0.07
***PCA_split_***	0.15 [ 0.00, 0.54]	<0.01*	0.24 [−0.19, 0.46]	<0.01*	−0.20 [−0.58, −0.05]	<0.01*
***ICA*** **+ ** ***PCA_split_***	0.19 [ 0.01, 0.54]	<0.01*	0.22 [−0.19, 0.62]	<0.01*	−0.25 [−0.72, 0.04]	<0.01*
***PCA_full_*** ** + ** ***PCA_split_***	0.25 [−0.14, 0.74]	<0.01*	0.28 [−0.19, 0.64]	<0.01*	−0.39 [−0.79, 0.20]	<0.01*

For *weak* contrast (TaskB vs. TaskA), model performance is shown for different subspace estimation methods, relative to full-dimensionality data (i.e. retaining all PCs). The median, [minimum, maximum] changes are shown for prediction (**Δ** *P*), reproducibility (**Δ** *R*) and distance **Δ** *D* from (*P* = 1,*R* = 1), over all single-subject results. Significance is given by Wilcoxon tests, with * indicating significant improvement. We show results for combinations of **ICA** = MELODIC subspace estimation, **PCA_split_** = optimized PC subspace on each data split-half, and **PCA_full_** = retaining 35% of PCs from the full data matrix. Note that (**PCA_full_**+**PCA_split_**) is the subspace selection method used for the rest of the manuscript. Results are shown for optimal fixed preprocessing: motion correction and 4^th^-order detrending.

### Individual-Subject Optimization of Experimental Data

**Figure 5:**
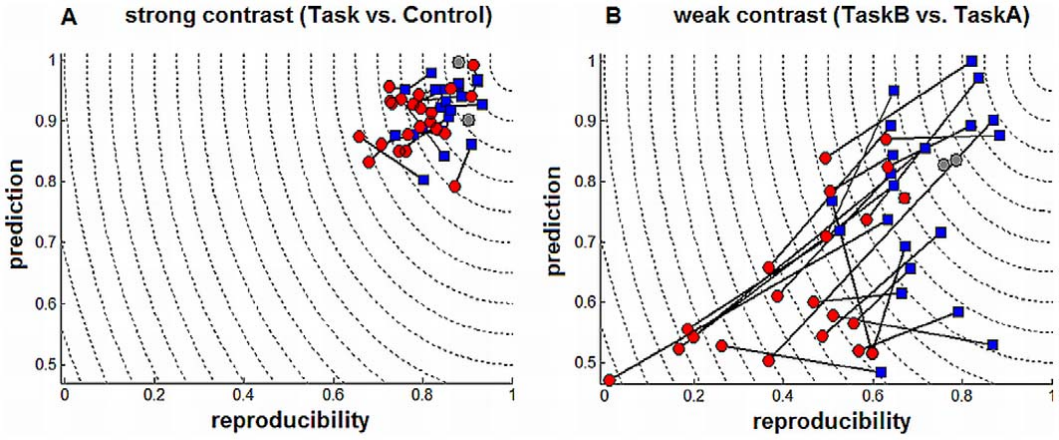
Effects of individual subject optimization on model performance. Prediction and reproducibility are plotted for the optimal fixed pipeline (red) and individually optimized pipeline (blue) of each subject, connected by a solid line. Performance metrics are plotted for (A) *strong* (Task vs. Control) and (B) *weak* (TaskB vs. TaskA) task contrasts. For *strong* and *weak* contrasts, optimal fixed pipelines are {ICA_M_,MC,DET2} and {ICA_M_,DET4}, respectively. Subjects with no change in pipeline are coloured in grey.

The (*P*,*R*) effect of *IND* preprocessing, which selects a heterogeneous set of pipelines distinct from *FIX*, is shown in Figure 5. Individual-subject (*P*,*R*) values are plotted for *FIX* (red circles) and *IND* (blue squares). For both contrasts, only 2 subjects were optimized with *FIX* preprocessing (e.g. showing no change; gray circles), all others had pipelines that reduced *D*(*P*,*R*). As may be expected, *strong* contrast results (Fig. 5(A)) had higher mean and lower variability in (*P*,*R*) than *weak* contrast (Fig. 5(B)). The *strong* and *weak* contrasts had respective mean *R* of 0.823 (range 0.657 to 0.994) and 0.593 (range 0.010 to 0.883), and respective mean *P* of 0.912 (range 0.792 to 0.997) and 0.711 (range 0.486 to 0.999), measured by averaging across both *FIX* and *IND* pipelines.

Individual optimization of *strong* task contrast generally improved *R*, with mean change 0.0564±0.0480 (21/24 subjects improved; *p*<0.01, paired Wilcoxon test), as well as *P*, with mean change 0.0132±0.0370 (18/24 subjects improved; *p* = 0.01). The *weak*-contrast results generally showed greater range of improvement in both metrics, with mean change in *R* of 0.242±0.180 (20/24 subjects improved; *p*<0.01, paired Wilcoxon test), and mean change in *P* of 0.138±0.125 (19/24 subjects improved; *p* = 0.01). For the *weaker* contrast results are thus more sensitive to *IND* optimization, for both prediction and reproducibility metrics.

**Figure 6:**
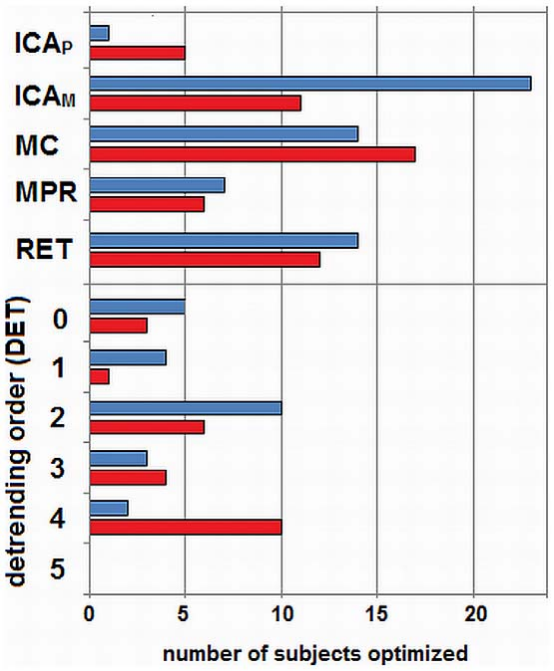
Frequency of preprocessing steps, for individually-optimized pipelines. The plot shows number of subjects (out of 24) optimized with each preprocessing step, under individual-subject optimization, for (blue) *strong* (Task vs Control) and (red) *weak* (Task B vs Task A) contrasts. Tested preprocessing steps include ICA with PESTICA (**ICA_P_**) and MELODIC (**ICA_M_**) algorithms, motion correction (**MC**), motion parameter regression (**MPR**), physiological noise correction via RETROICOR (**RET**) and detrending with Legendre polynomials of order 0–5 (**DET0**–**5**).

Figure 6 summarizes the number of subjects optimized with each preprocessing step for *IND*. As with fixed preprocessing, we observed trends specific to the two task contrasts. For the strong contrast (blue bars), 23/24 subjects optimized with ICA_M_, 17/24 optimized with MC and RET, and 10/24 optimized with DET2, indicating that these tended to be the most important pipeline steps. However, there remains significant heterogeneity in the optimal detrending order, and a subset of subjects required MPR and DET0–4, although only one subject was optimized with ICA_P_. For *weak* contrast (red bars), we observed two major changes in pipeline trends. First, we see changes in ICA, with ICA_M_ becoming less important (only 11/24 subjects optimized) and ICA_P_ now optimizing 5/24 subjects. Second, there was a shift in detrending order, with the higher-order DET4 becoming the most consistent optimum (10/24 subjects). Trends in RET, MPR and MC did not show marked differences between task contrasts.

**Figure 7:**
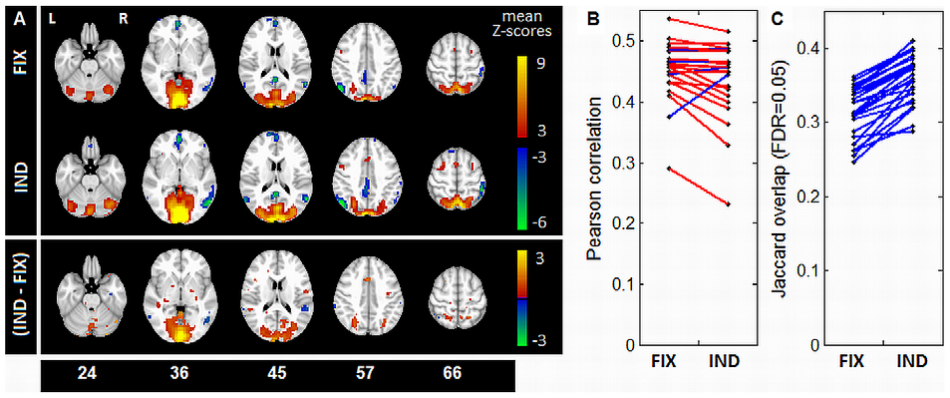
Effects of pipeline optimization on group-level activation, *strong* contrast. Activation structure is shown for *strong* (Task vs. Control) contrast, under fixed (**FIX**) and individually-optimized (**IND**) preprocessing. (A) mean Z-scored activation maps for **FIX** and **IND** (top), and mean Z-score change (bottom), with significance given by bootstrap estimation (1000 iterations), thresholded at False-Discovery Rate (FDR) 0.05. (B) between-subject SPM correlations, for both **FIX** and **IND** pipeline sets. (C) between-subject Jaccard activation overlap, for voxels significant at FDR = 0.05. For (B–C), each point represents mean correlation/overlap of one subject with all others; blue lines show an increase in correlation/overlap with **IND**, and red lines show a decrease.

Figure 7(A) plots the mean SPM for both *FIX* and *IND* pipelines, for *strong* contrast results. This included positive activations in the cerebellum (slice 24), visual cortex (slices 36–45), cuneus and superior occipital lobes (slice 57), precuneus and superior parietal lobes (slice 66), supplementary motor area (SMA) and precentral gyri (slices 57,66). Negative activations included ventral anterior cingulate cortex (vACC) and right inferior temporal lobe (ITL) (slice 36), superior medial-frontal gyrus, middle temporal lobes (MTL) and posterior cingulate cortex (PCC) (slice 45–46), angular gyri (slice 57) and right inferior parietal lobe (slice 66). The mean difference plot (Fig. 7(A), bottom) shows that although both pipelines tended to identify similar regions of activation, *IND* shows significant mean Z-score increases in posterior task-positive activations, along with SMA and right-side ITL and MTL. Fig.s 7(B–C) show that *IND* optimization decreased correlation between subject SPMs for 18/24 subjects (red lines), but increased mean activation overlap (FDR = 0.05), for all subjects (blue lines), significant at *p*<0.01 (paired Wilcoxon).

**Figure 8:**
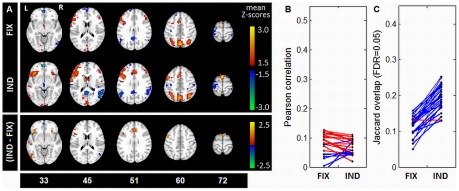
Effects of pipeline optimization on group-level activation, *weak* contrast. Activation structure is shown for *weak* (TaskB vs. TaskA) contrast, under fixed (**FIX**) and individuallyoptimized (**IND**) preprocessing. (A) mean Z-scored activation maps for **FIX** and **IND** (top), and mean Z-score change (bottom), with significance given by bootstrap estimation (1000 iterations), thresholded at False-Discovery Rate (FDR) 0.05. (B) between -subject SPM correlations, for both **FIX** and **IND** pipeline sets; note that one point with correlation −0.014 was omitted from the displayed range for clarity. (C) between -subject Jaccard activation overlap, for voxels significant at FDR = 0.05. For (B–C), each point represents mean correlation/overlap of one subject with all others; blue lines show an increase in correlation/overlap with **IND**, and red lines show a decrease.

Figure 8(A) compares mean SPMs for *FIX* and *IND* for the *weak* task contrast. Regions of largest positive signal include predominantly left-side activations of inferior orbitofrontal (slice 33), inferior frontal (slice 45,51), caudate (slice 45), anterior cingulate (slice 51), precentral gyrus (slice 60) and SMA (slices 60,72). Task-negative activations appear in vACC (slice 33), PCC and MTL (slices 45,51) and postcentral gyri (slices 60,72). Regions of significant *IND*-*FIX* change (Fig. 8(A) bottom) were more sparse, likely due to elevated intersubect heterogeneity in absolute Z-scores (comparing intersubject correlations of Fig. 7(B) and Fig. 8(B)). However, significant task-positive increases occurred in inferior orbitofrontal (slice 33), left inferior frontal gyrus (slices 45,51), anterior cingulate (slice 51), SMA (slice 72) and precentral gyrus (slice 60). Significant task-negative changes were sparser, appearing in vACC (slice 33), right MTL (slice 45) and right postcentral gyrus (slice 60). Fig. 8(B–C) again shows that *IND* optimization consistently decreased inter-subject correlations (18/24 subjects), but 23/24 subjects showed increased mean activation overlap (FDR = 0.05), significant at *p*<0.01 (paired Wilcoxon). The inter-subject correlations and overlaps were consistently lower for *weak* contrast than *strong* (Fig. 8(B–C) vs. Fig. 7(B–C)), irrespective of pipeline optimization.

## Discussion

This paper presents the first comprehensive study of the interaction between multiple steps of the fMRI experimental pipeline, including task contrast, preprocessing pipeline, and heterogeneity of subject effects. The presented results show that while individual subject optimization of preprocessing significantly affects (*P*,*R*) of fMRI analyses, there are also consistent interactions between other components of the experimental pipeline. In particular, subject-specific pipeline optimization has a greater effect on (*P*,*R*) for *weaker* cognitive contrasts, which may require distinct preprocessing sets, relative to *stronger* contrast. It is thus important to consider the data-analysis pipeline as a whole, when choosing preprocessing steps and models for optimal fMRI analyses.

### Testing bias of individual subject optimization

In the initial simulation analyses, we demonstrated that individual subject optimization does not significantly increase model bias, relative to fixed preprocessing. This provides the first validation of individual subject pipeline optimization using a measure of “ground truth”, although such optimization has already been applied in a number of experimental contexts (e.g. [8], [18], [19]). The optimization procedure is not significantly biased, because we are jointly maximizing prediction and reproducibility metrics, instead of the more common target of voxel signal strength – the latter carries the risk of circular analysis (see Kriegeskorte *et al.*, [55]), in which the experimenter tunes the pipeline to maximize Z-scores (for example), fitting the model to experimental noise in the process. The presented results demonstrate that the common approach of fixed preprocessing in the fMRI literature is not intrinsically more robust to bias than individual subject optimization, even in the most limiting case of independent, identically-distributed signal and noise. In fact, PC subspace optimization significantly improves within-sample signal detection in weaker CNR = 0.3 data, even for a Gaussian, identically-distributed set of data samples.

### Fixed-Pipeline Optimization of Experimental Data

The fixed-pipeline procedure provided information on the effects of preprocessing choice across subjects in a given task design, and constitutes a statistically rigorous method of determining which preprocessing choices are most important. For example, in *strong* contrast, ICA_M_ and MC are important to optimize (*P*, *R*), whereas for the *weaker* contrast, a more heterogeneous set of pipelines was observed (compare Tables 2 and 3). By comparison, Churchill *et al.* [17] also reported significant ranking of fixed pipelines in which MC is typically included, for *strong* contrast without an ICA denoising step, However, [17] also found DET2 to be most important for fixed-pipeline optimization, which is not the case in the current results. The inclusion of ICA_M_ thus interacts with detrending, increasing the heterogeneity of its effect across subjects. Our fixed-pipeline results also demonstrated that pipelines with comparable (*P*, *R*) exhibited significantly different activation patterns; DISTATIS was effective at clustering pipelines based on shared spatial structure. These results are consistent with [17], which demonstrated that although pipeline (*P*,*R*) is correlated with SPM pattern, not all significant differences in pipeline SPMs are captured by (*P*,*R*) metrics.

The optimal fixed pipelines also vary significantly as a function of task contrast. For example, MELODIC denoising is typically optimal for the *stronger* contrasts, with more heterogeneous effects in the *weaker* case. This indicates that MELODIC is unable to effectively separate signal and noise for many subjects, mixing TaskB vs. TaskA signal into the nominally structured artifact ICs. This is an expected risk, as artifact sources, including macrovascular flow, respiration and motion, often correlate spatially and temporally with BOLD response [56], [57]. In addition, it has been recently shown that the initial probabilistic PCA dimensionality estimator used in MELODIC is suboptimal for network pattern detection [43]. These may also be issues for the *weak* contrast, due to the signal's smaller variance and less stable distribution (supported by generally lower (P,R) values), which the ICA model appears unable to separate based on independence measures. This may be contrasted with PESTICA, which becomes marginally more important for *weak* contrast; this method estimates ICs using *a priori* spatiotemporal constraints. It is rarely an optimal step, even for *weak* contrast, possibly because PESTICA uses a fixed set of 6 regressors, which estimates a fixed-dimensional noise subspace. This is known to be sub-optimal noise estimation, as the dimensionality of physiological noise varies from subject to subject [58]; the flexibility of PESTICA may be significantly improved by individual dimensionality optimization. The two ICA methods illustrate important tradeoffs in data-driven modelling, which may vary from *a priori* unconstrained but dimensionally-flexible (MELODIC) to physiological-based priors but fixed dimensionality estimation (PESTICA).

However, one of the challenges of comparing ICA models is that they often differ considerably in implementation, making it difficult to isolate the causes of differing performance. In addition to dimensionality and spatial constraints, MELODIC and PESTICA vary in such parameters as initial subspace estimators (Probabilistic PCA vs. PCA), dimension of component independence (spatial vs. temporal) and algorithmic convergence criteria [28], [52]. Further analyses are required to definitively establish which parameters are driving the contrast-dependent tradeoff between MELODIC and PESTICA models.

Additionally, although ICA denoising is generally beneficial for the control of artifacts, MELODIC proved to be a sub-optimal procedure for estimating the signal subspace. This is consistent with prior analyses of Yourganov et al. [43], who found that ICA detection of brain networks underperforms, relative to linear discriminant methods. This may be partly due to the PCA-based dimensionality estimation method used in MELODIC. This package uses optimization of Bayes' evidence to estimate intrinsic dimensionality, which has also been shown to produce sub-optimal dimensionality estimates [43]; this suggests that optimizing the ICA subspace using a data-driven step-up procedure optimized on (P,R), as with PCA, may potentially improve this model. Nonetheless, results indicate that optimizing the subspace using PCA generally allows for more predictive and reproducible signal subspace estimation; this is beneficial, given the relative computational efficiency and consistency of the estimated subspace for PCA, over ICA algorithms.

For fixed preprocessing, RET is increasingly important for *weaker* contrast, indicating a more consistently positive impact. This partially validates the trend in fMRI literature, of performing more extensive physiological corrections for weaker-signal analyses (e.g. compare [33], [34] to [35], [37]). This is likely due to changes in relative variance of signal and noise, to which multivariate analysis models are particularly sensitive. As we transition from large, spatially localized BOLD signal changes (*strong* contrast; Fig. 3) to smaller, distributed changes (*weak*-contrast and resting state dynamics; Fig. 4), physiological variance likely becomes increasingly larger than BOLD-related variance. This requires more extensive denoising to sufficiently reduce noise confounds for signal detection. These results are consistent with prior findings of [17], in which subjects with greater proportionate head motion were optimized with more extensive RET and MPR preprocessing. Interestingly, we do not observe an increase in RET (or MPR) for individually-optimized *weak* contrast results; this indicates that selecting MELODIC or PESTICA on a subject-specific basis may be effective for controlling contrast-dependent increases in noise variance. However, many subjects still require MPR and RET to optimize (*P*, *R*), further demonstrating that ICA alone is insufficient to minimize residual motion and physiological artifact, for many subjects.

For *weaker* contrast, we also found that MC is less frequently selected as an optimal fixed-pipeline step. However, given that the majority of individually optimized subjects require MC (17/24 subjects) after controlling for task-coupled motion artifact as outlined in Text S4, it is likely that fixed pipelines without MC (and any other motion correction procedure) are also reinforcing such artifact. However, the last fixed optimization step (selecting the pipeline that maximized group activation overlap) without adjusting for individual task-coupled motion effects appears to also control for this issue; in the weak contrast, we discard the {RET,DET4} pipeline (with an extensive activation “rim” along brain edges, shown in Fig. 4(B) (top), and no motion correction method), in favour of {ICA_M_,DET4} (motion denoising performed within MELODIC).

### Individual-Subject Optimization of Experimental Data

The results of individual-subject optimization reveal further trends in preprocessing effects. Under individual-subject optimization, regression methods become consistently more important for weaker and more distributed brain signal (*weak* contrast), having a greater impact on (*P*, *R*) metrics. In particular, Fig. 5(B) shows results for subjects with near zero signal detection under *FIX* (that is, near (*P*≈0.5, *R* = 0)) improved beyond the majority of fixed-pipeline results, under *IND* optimization. This demonstrates that subjects that would otherwise be discarded due to excessive noise/overly weak BOLD signal may simply be limited by suboptimal preprocessing choices. These results have implications for analyses across age groups and clinical populations. In these cases of weak or atypical BOLD signal [59], individual optimization may prove increasingly important for robust measurements.

Examining trends in optimal preprocessing (Fig. 6), we note that DET2 is most consistently optimal for *strong* contrast, but more heterogeneous for *weak*, with DET4 being more consistently optimal; this suggests increasing between-subject heterogeneity in the structure and impact of low-frequency drift effects. Note that AFNI's heuristic model of low-frequency drift (used, in the *3dDeconvolve* GLM model [49]) recommends DET2 for this dataset; this model is thus an effective predictor of optimal fixed *strong*-contrast detrending, but does not generalize to *weak*-contrast or individual-subject heterogeneity.

In addition to validating individual-subject pipeline optimization using simulation analyses, we performed group-level comparisons of optimized rSPM(Z)s. These results demonstrate that individual subject optimization extracts significant, consistent loci of greatest activation in grey matter. However, individual optimization also generally decreases correlations between subject rSPM(Z)s. This indicates that we are increasing the variability of voxel Z-scores between subjects, although the regions of highest activation increase in spatial consistency. It has been previously demonstrated that loci of highest mean activation show greatest inter-subject signal variability [17], [60]. Furthermore, Churchill et al. [17] have directly shown that, for a reduced pipeline set, individual-subject optimization increases both Z-score variability and between-subject overlap of significantly activated voxels.

Regarding analyses of Trails-Making Test rSPM(Z) patterns, the present work identified a set of reproducible activations across different preprocessing methods. These activations are also consistent with prior literature on the Trails-Making Test, including dorsolateral prefrontal activations and negative activation consistent with the Default Mode Network [17], [41], [48], a known predictor of cognitive health and aging (e.g. [22]), as well as occipital/parietal activations specific to *Task* vs. *Control* [17], and superior-frontal activations for TaskB vs. TaskA contrast [41], [48].

### Further Implications

An important outcome of the work is that for strong, focal activations, *FIX* preprocessing may provide near-optimal results. This validates the practises of standard functional neuroimaging in experiments with strong signal-to-noise and focal activation, such as those involving primary sensory processing and motor behaviour. However, for cases of weaker fMRI signal, involving subtle cognitive contrasts, preprocessing choices must be more carefully evaluated. In these cases, analysis of distributed networks should involve careful testing of pipeline choices - which is not a common practice. The presented results provide an initial framework for such testing, demonstrating which preprocessing steps have the most critical influence on experimental pipeline conditions and subject data. Future work will involve examining the effects of other parts of the experimental pipeline, including acquisition parameters, subject demographics (e.g. subject age and health), and analysis models (e.g. univariate vs. multivariate methods). Such pipeline characterization may allow the ultimate goal of *a priori* prediction of the optimal pipeline steps, in order to optimize signal detection.

## Supporting Information

Figure S1 **Procedure for estimating a reproducible, Z-scored SPM (rSPM(Z)).** (A) the dataset is temporally split into 2 halves, and analysis performed on each split-half, generating 2 independent SPMs. (B) a 2D scatterplot is produced of split1/2 voxel values; for example, voxel values V_1_ and V_2_ of (A) produce a point with coordinates (V_1_,V_2_) in the scatterplot. A PCA of the scatterplot gives orthogonal signal and noise axes (1^st^ and 2^nd^ PCs, respectively). (C) histograms of voxel signal, projected onto signal/noise axes; we also mark the standard deviation (SD) of the noise axis scatter. (D) The rSPM(Z) is computed by normalizing the signal-axis values by SD(noise axis), then mapping the values back to their respective brain locations.
(TIF)

Figure S2 **Critical-difference diagram for a subset of 6 preprocessing pipelines.** The horizontal axis is median pipeline rank, computed over all subjects, based on distance from (prediction = 1, reproducibility = 1); the highest-ranked (optimal) pipeline is {ICA_M_, MC}. The Critical-Difference (CD) interval based on a Nemenyi test is also shown (α = 0.05). Pipelines with separation <CD are not significantly different (connected by blue/gray bars). Pipeline {MC} is not significantly worse than the highest-ranked {ICA_M_, MC} (blue bar), and is thus also considered optimal. A fixed polynomial detrending order of 2 was held for all pipelines. Preprocessing steps are denoted: MC = motion correction, MPR = motion parameter regression, ICA_P_/ICA_M_ = ICA denoising with PESTICA/MELODIC.
(TIF)

Text S1 **Criteria for Manual Selection of ICA components.**
(DOC)

Text S2 **Model optimization with Prediction and Reproducibility.**
(DOC)

Text S3 **Details of the Fixed-Pipeline Optimization Procedure.**
(DOC)

Text S4 **Diagnosing Task-Coupled Motion Artifact.**
(DOC)
