# Supplementary figures and images for: Optimizing Preprocessing and Analysis Pipelines for Single-Subject fMRI: 2. Interactions with ICA, PCA, Task Contrast and Inter-Subject Heterogeneity
Source: PLoS One. 2012 Feb 27;7(2):e31147. doi: 10.1371/journal.pone.0031147 (PMC3288007; doi:10.1371/journal.pone.0031147)

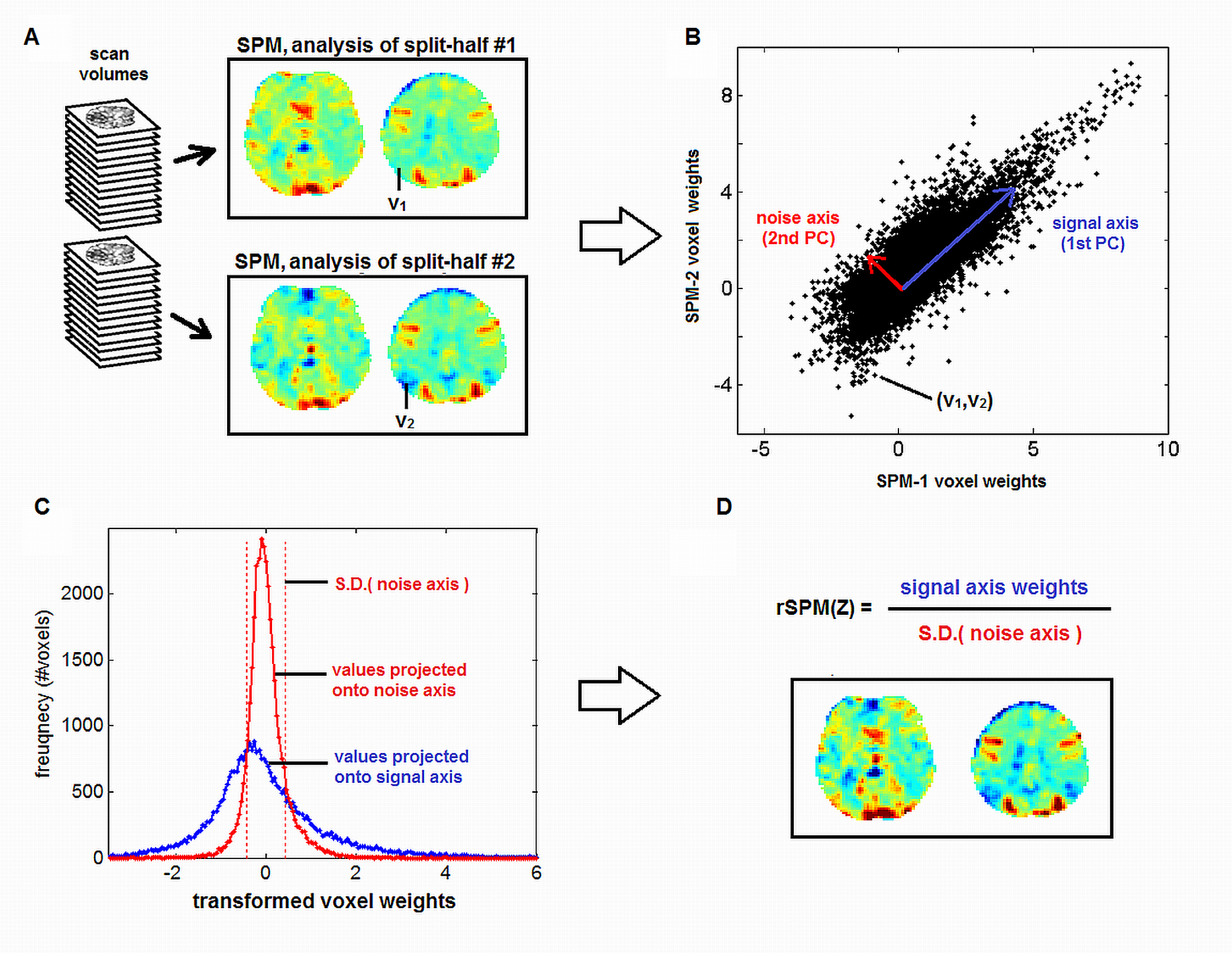

Supplement: Figure S1 — Procedure for estimating a reproducible, Z-scored SPM (rSPM(Z)). (A) the dataset is temporally split into 2 halves, and analysis performed on each split-half, generating 2 independent SPMs. (B) a 2D scatterplot is produced of split1/2 voxel values; for example, voxel values V1 and V2 of (A) produce a point with coordinates (V1,V2) in the scatterplot. A PCA of the scatterplot gives orthogonal signal and noise axes (1st and 2nd PCs, respectively). (C) histograms of voxel signal, projected onto signal/noise axes; we also mark the standard deviation (SD) of the noise axis scatter. (D) The rSPM(Z) is computed by normalizing the signal-axis values by SD(noise axis), then mapping the values back to their respective brain locations. (TIF) [file pone.0031147.s001.tif]

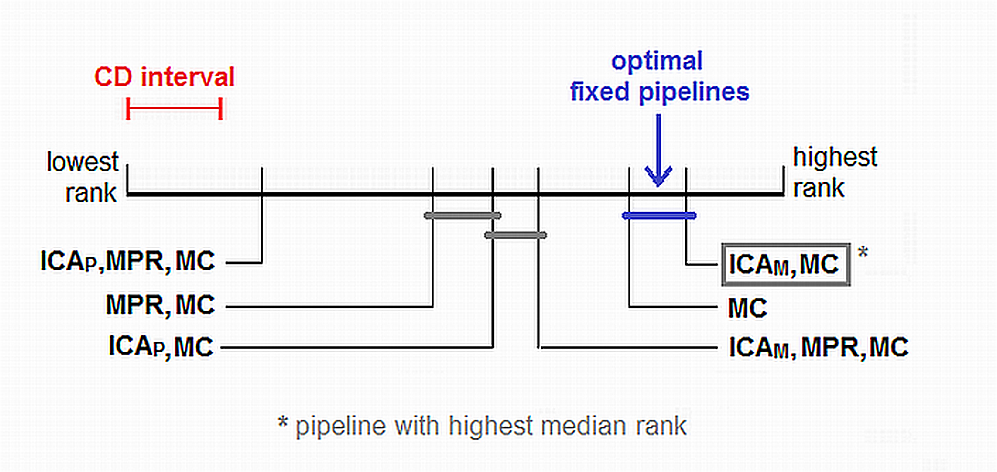

Supplement: Figure S2 — Critical-difference diagram for a subset of 6 preprocessing pipelines. The horizontal axis is median pipeline rank, computed over all subjects, based on distance from (prediction = 1, reproducibility = 1); the highest-ranked (optimal) pipeline is {ICAM, MC}. The Critical-Difference (CD) interval based on a Nemenyi test is also shown (α = 0.05). Pipelines with separation <CD are not significantly different (connected by blue/gray bars). Pipeline {MC} is not significantly worse than the highest-ranked {ICAM, MC} (blue bar), and is thus also considered optimal. A fixed polynomial detrending order of 2 was held for all pipelines. Preprocessing steps are denoted: MC = motion correction, MPR = motion parameter regression, ICAP/ICAM = ICA denoising with PESTICA/MELODIC. (TIF) [file pone.0031147.s002.tif]
