# Supplementary material for: Optimizing Preprocessing and Analysis Pipelines for Single-Subject fMRI: 2. Interactions with ICA, PCA, Task Contrast and Inter-Subject Heterogeneity
Source: PLoS One. 2012 Feb 27;7(2):e31147. doi: 10.1371/journal.pone.0031147 (PMC3288007; doi:10.1371/journal.pone.0031147)
Supplement: Text S1 — Criteria for Manual Selection of ICA components. (DOC) [file pone.0031147.s003.doc]

**Text S1: Criteria for Manual Selection of ICA components**

For component selection, we examined the set of Independent Components (ICs) identified as significant by MELOLDIC’s Bayesian estimation process [52]. The ICs were identified as artifactual, based on well-known spatiotemporal characteristics of physiological and motion artifact:

**Physiological artifact**: spatial markers include signal predominantly located in ventricles and sinuses, near regions of dense macrovasculature (e.g. the Circle of Willis), or restricted to the brainstem. Temporal identifiers included rapid oscillation due to bloodflow pulsatility; in these cases, spectral peaks formed predominantly above a threshold of 0.125 Hz.

**Motion artifact**: spatial markers include bands of activation about the brain edge, strong signal localized to the top/bottom of the brain, and activations that cover the full extent of the brain, particularly in alternating slices (reflecting large inferior-superior movement coupled with interleaved slice acquisition). Temporal indicators include approximately linear-quadratic drift patterns, and timecourses that are dominated by isolated “spikes” or discontinuities in the timecourse.

The selection of artifact ICs was generally conservative; if any spatial maps contained signal localized in grey matter regions of potential activation, based on prior observations of the mean subject SPMs, or timecourses evidenced transient correlation with the task design, the components were retained as potentially containing BOLD signal.
