# Supplementary material for: Optimizing Preprocessing and Analysis Pipelines for Single-Subject fMRI: 2. Interactions with ICA, PCA, Task Contrast and Inter-Subject Heterogeneity
Source: PLoS One. 2012 Feb 27;7(2):e31147. doi: 10.1371/journal.pone.0031147 (PMC3288007; doi:10.1371/journal.pone.0031147)
Supplement: Text S2 — Model optimization with Prediction and Reproducibility. (DOC) [file pone.0031147.s004.doc]

**Text S2: Model optimization with Prediction and Reproducibility**

The two data-driven metrics used to optimize pipeline choice are spatial reproducibility (*R*) and temporal prediction accuracy (*P*), estimated in the NPAIRS split-half resampling framework [38]. This section discusses details of these metrics and the importance of jointly optimizing (*P*, *R*).

Reproducibility

This metric estimates how consistent extracted SPMs’ voxel values are for a given data analysis pipeline, across (pseudo-)independently acquired datasets. For this measure, we compute an SPM independently on each of 2 data split halves from the dataset of interest (e.g., a single subject’s fMRI run, or independent split-half groups of a multi-subject group analysis); we then produce a scatter plot, where each point represents a single voxel’s paired (split-1,split-2) SPM signal values (Figure S1(a)). The Pearson correlation of these 2 SPM vectors provides a global measure of SPM reproducibility, and it is monotonically related to the global Signal-to-Noise (gSNR) of the image, via equation [39, 43]:

$gSNR=\sqrt{\frac{2R}{1-R}}$ (S1)

We may also use this framework to obtain a reproducible Z-scored SPM that maximizes estimated signal. If each split-half SPM has voxel values normalized by their spatial standard deviation, the major axis (1^st^ component of a PCA on the scatterplot, accounting for most variance) is along the line of identity. This is the signal axis (Fig. S1(b), blue arrow), and we project pairwise voxel values onto this line, in order to obtain the most-reproducible signal pattern (Fig. S1(c), blue curve). For SPMs in vector form *X*_1_ and *X*_2_, the transformed vector *X*_sig_ is:

(S2)

The minor axis (i.e. 2^nd^ component of a PCA on the scatter-plot), which is orthogonal to the line of identity, forms the “noise axis” (Fig. S1(b), red arrow). The projection onto this axis measures non-reproducible signal at each voxel (Fig. S1(c), red curve), giving noise vector *X*_noi_:

(S3)

The standard deviation of *X*_noi_ provides a global estimate of SPM noise, which we use to normalize *X*_sig_, to obtain reproducible, Z-scored SPM (rSPM(Z); see Fig. S1(d)):

$rSPM(Z)=\frac{X_{sig}}{std\left(X_{noi}\right)}$ (S4)

These methods provide our measure of model reproducibility, as well as an rSPM(Z) that maximizes the reproducible spatial signal across split-halves of the dataset of interest. Note that this measure is robust to dataset heterogeneity as the more unequal the split halves are the more rSPM(Z) is downweighted through division by std(*X*_noi_) in (Eqtn. S4).

Prediction

This metric quantifies how well a model generalizes to independent results; prediction requires that we build our classifier model on a *training* dataset, after which we measure its ability to correctly classify scans in an independent *test* set. After building the *training* classifier with model parameters θ, we estimate conditional probability *p*(*C*_k_ | *X*; θ) of correct assignment to class *C_k_* (1 < k < K), for *test* datapoint *X*. In this framework, we attempt to predict cognitive state (class *C*_k_) based on brain measures (fMRI image vector *X*; a “mind-reading” model), defined by the Bayes posterior probability rule:

$p(C_{k}|X;\theta)=\frac{p(X|C_{k};\theta)\cdot p(C_{k})}{p(X)}$ (S5)

This is the generalized form of our prediction measurement; we replace unknown, fixed prior probability *p*(*X*) with a normalizing constant, to ensure that the sum across posterior class probabilities is unity:

$p(C_{k}|X;\theta)=\frac{p(X|C_{k};\theta)\cdot p(C_{k})}{\sum_{i=1}^{K} p(X|C_{i};\theta)\cdot p(C_{i})}$ (S6)

Prior class probability *p*(*C*_k_) is the proportion of scans in *C*_k_ (e.g. *p*=0.5 for the TrailsA/TrailsB classes in our *weak*-contrast analyses); conditional probability *p*(*X* | *C*_k_; θ) is dependent on the chosen analysis model. For our current results, we performed Penalized Discriminant Analysis (Linear Discriminant Analysis on an optimized Principal Component subspace); for details of this classifier, refer to [63], and for related models used in an NPAIRS (P,R) framework see [64]. The PDA model provides a linear transformation matrix *L*_train_ into the *training* discriminant subspace, where *L*_train_ is normalized so that *training* variance in this subspace is unity. In this case, the conditional posterior probability of *X* for known class *C*_k_ reduces to the multivariate normal function:

$p(X|C_{k};\theta)=\frac{1}{\sqrt{2\pi}}\exp\left(-\frac{1}{2}\left\|L_{train}^{T}\left(X-\bar{X}^{(k)}_{train}\right)\right\|^{2}\right)$ (S7)

where $\bar{X}^{(k)}_{train}$ is the training mean of data from class *C*_k_. This allows us to estimate posterior probability *p*(*C*_k_ | *X*; θ) for each scan. The mean *p*(*C*_k_ | *X*; θ) is computed over all scans in a given *test* split; afterwards, we compute the median of these mean values (across all *test* split-halves), as a measure robust to outlier splits, to produce a single prediction measure *P*.

Joint Optimization of Prediction and Reproducibility

Although optimizing model prediction and reproducibility are goals for any neuroscientific experiment, it is often difficult if not impossible to simultaneously optimize both metrics in highly ill-posed neuroimaging datasets with many more variables (e.g. voxels) compared with observations (e.g. scans). This is due to prediction and reproducibility representing important tradeoffs in model parameterization; it is thus also generally undesirable to strictly optimize on one metric [64]. Models driven by *R* optimization tend to have more stable SPM patterns, but are often less sensitive to the stimulus-coupled brain response (i.e. poor generalizability). As a simple illustration of this issue, an analysis model that ignores the data input and outputs a fixed SPM pattern will be perfectly reproducible (*R* = 1), but has no ability to predict brain state. By contrast, models driven by *P* will be highly predictive of stimulus condition, but tend to extract unstable (non-reproducible) discriminant patterns. As an example, a discriminant model that is driven by a small number of the most task-coupled brain voxels may be highly predictive of class structure (*P* ≈ 1), but will have low reproducibility, as all other voxels values will tend to be more variable to random between splits.

Standard analysis models and experimental data rarely provide such extreme results. However, it has been previously shown that the choice of optimization criteria significantly alters results, potentially identifying different or only partial brain networks, with varying signal strengths and spatial extents [19, 43, 64]. The joint optimization of (P, R), based on Euclidean distance from (P=1, R=1) provides an effective compromise between the two model parameterizations.
