# Supplementary material for: Optimizing Preprocessing and Analysis Pipelines for Single-Subject fMRI: 2. Interactions with ICA, PCA, Task Contrast and Inter-Subject Heterogeneity
Source: PLoS One. 2012 Feb 27;7(2):e31147. doi: 10.1371/journal.pone.0031147 (PMC3288007; doi:10.1371/journal.pone.0031147)
Supplement: Text S3 — Details of the Fixed-Pipeline Optimization Procedure. (DOC) [file pone.0031147.s005.doc]

**Text S3: Details of the Fixed-Pipeline Optimization Procedure**

In order to select the optimal fixed pipeline set (i.e. a single set of preprocessing choices applied to all subjects), for a group of *N* subjects with *K* preprocessing pipelines, we applied a 2-step selection procedure: (a) identify the set of optimal pipelines based on prediction and reproducibility (*P*, *R*) metrics, and (b) characterize this set of optimal pipelines, based on SPM spatial structure. This provides a principled, quantitative method to identify the most important preprocessing choices for a fixed pipeline. The details of these methods are as follows:

(a) Optimal Pipelines based on (P,R) Metrics

This step identifies the fixed pipeline set that most consistently minimizes Euclidean distance *D* from (*P*=1,*R*=1), for the *N* subjects. Because (*P*,*R*) distributions are heterogeneous across both subjects and pipelines, we employ a rank-normalization procedure, first demonstrated in Churchill et al. [17], to test for a significant ordering in pipeline performance:

1. For each of the *N* subjects, rank the pipelines 1-*K*, by their *D*(*P*, *R*) value, with smaller *D* and higher rank indicating better pipeline performance.
2. For each pipeline *k* (1 < *k* < *K*), compute the median ranking across all *N* subjects’ values, for a measure of relative performance, and identify the pipeline with highest median ranking, denoted *PPL*_opt_ .
3. Perform the Friedman multiple-treatment test [53]; this is the non-parametric analogue to repeated-measures ANOVA. This procedure tests for consistent differences in pipelines’ *D*-values (treatment response) across multiple subjects (samples). It is performed on the treatment rankings from step #2, which avoids any assumptions about the *D*-value distribution for a given subject.
4. If the Friedman test identifies a significant pipeline ordering, we performed a multiple-comparison Nemenyi test [53], based on the sum of ranks at each pipeline. It estimates the critical-difference (CD) interval: the range at which a difference between pipeline rank-sums is greater than expected, based on the standard error of the rank-sum distribution. Pipelines with rank-sum differences less than the CD interval are not significantly distinguishable.
5. We identify the set of *L* pipelines that are within the CD interval from the *PPL*_opt_ pipeline, at α=0.05, and thus not significantly worse than *PPL*_opt_.

This method is used to identify a set of *L* optimal fixed pipelines at 95% confidence, for a given set of subjects. Figure S2 shows an example of this median pipeline ranking, and 95% confidence interval, for a subset of pipelines.

(b) Characterizing Fixed Pipeline Spatial Structure

For the set of *L* pipelines with statistically indistinguishable, optimal (*P*, *R*) distributions, we then compared the spatial structure of the rSPM(Z)s, to determine if the pipelines produce significantly different SPM patterns. We used the three-way multidimensional scaling method of DISTATIS [54,55]. For this method, we measure the *L*x*L* correlation matrix of the *L* optimal pipeline SPMs, for each subject; this is used to estimate the most consistent pattern of SPM similarity across all *N* subjects. This technique allows us to (1) obtain a denoised estimate of the most common SPM similarity pattern, using PCA methods, and (2) perform Bootstrap resampling on the set of subject correlation matrices, in order to estimate the 95% confidence ellipses on our pattern of SPM similarity. For *L* fixed pipelines and *N* subjects, the procedure is:

1. For each subject *n* (1 < *n* < *N*), compute the *L*x*L* correlation matrix *S*_n_ between the *L* pipeline SPMs, with row- and column-centering. This provides *N* samples, from which we attempt to estimate the “most consistent” SPM correlation pattern.
2. Compute pairwise similarity between each of the *S*_n_ matrices (1 < *n* < *N*), by measuring the R_V_ coefficient of matrix similarity between each pair of *S*_n_, to form the *N*x*N* inter-subject similarity matrix ***C***.
3. We now estimate the strongest average *S*-matrix pattern (SPM correlation pattern) expressed across all subjects, by performing PCA on the matrix ***C***. The first eigenvector represents a set of coefficients α_n_, that weight how similar each *S*_n_ (and thus the *n*^th^ subject) is to the strongest common *S*-matrix pattern. We then compute this strongest common pattern (the *compromise*), as $S_{+}=\sum_{i=1}^{N} \alpha_{i}S_{i}$, which expresses the most common *L*x*L* pattern of pipeline SPM correlations.
4. We now represent the similarity structure of ***S***_+_ in a manner that maximizes the variance between pipeline SPMs, by performing PCA on ***S***_+_ (i.e. an eigenvalue decomposition), and projecting ***S***_+_ into the new basis space. This represents each pipeline’s SPM as a point in (*L*-1)-dimensional PCA space. The Euclidean distance between any two pipelines’ points in the (*L*-1)-dimensional space measures the difference between pipeline SPMs, with points that are closer together indicating more similar activation maps.
5. We also produced confidence estimates on the pipeline points, by Bootstrap resampling of the set of correlation matrices *S*_n_ (1 < *n* < *N*), to generate repeated estimates of ***S*_+_** (Steps 2-3), each of which were then projected into the original PC space, with 1000 iterations performed. A 95% confidence ellipse was then drawn around the Bootstrapped set of 1000 PC-space coordinates, for each pipeline.

This method provides an empirical, nonparametric method to cluster pipeline SPMs; pipeline groups with overlapped 95% confidence ellipses produce SPMs that are not significantly distinguishable. This allows us to reduce the number of necessary pipeline choices, as we need only select one representative from each cluster (since all others are not significantly different in either (P,R) or spatial pattern).

(c) Maximizing Between-Subject Overlap

We can then apply a final selection criterion on the greatly reduced set of pipeline choices. For the current paper, we chose the pipeline maximizing average Jaccard overlap between subject SPMs (the number voxels active in *both* SPMs (the intersection), divided by the number of voxels active in *at least one* SPM (the union)), for a False-Discovery Rate (FDR) = 0.05 threshold. This measure is chosen under the assumption that pipeline optimization reinforces activations in brain regions that are spatially consistent across all subjects.
