# Supplementary material for: Optimizing Preprocessing and Analysis Pipelines for Single-Subject fMRI: 2. Interactions with ICA, PCA, Task Contrast and Inter-Subject Heterogeneity
Source: PLoS One. 2012 Feb 27;7(2):e31147. doi: 10.1371/journal.pone.0031147 (PMC3288007; doi:10.1371/journal.pone.0031147)
Supplement: Text S4 — Diagnosing Task-Coupled Motion Artifact. (DOC) [file pone.0031147.s006.doc]

**Text S4:** **Diagnosing Task-Coupled Motion Artifact**

One of the challenges of optimization based on prediction accuracy and spatial reproducibility (*P*,*R*), is that head motion may also be correlated with stimuli, and induce signal change in spatially consistent brain regions. These effects are not only difficult to separate from BOLD signal, but may drive model optimization if BOLD signal has comparatively low (*P*,*R*). In order to detect when model optimization is driven by task-coupled motion (TCM), we have established a test that identifies TCM by the spatial distribution of signal in the SPMs:

1. For a set of *K* subjects, identify the optimal pipeline with motion correction (MC) held fixed for each subject. We assume that MC provides a minimum level of control against head motion; no pipeline choice that excludes MC should significantly increase the amount of motion artifact.
2. For each subject (1 < *k* < *K*):
   1. Estimate edge artifact structure: Take the 4D timeseries of the optimized pipeline, and compute the first-order spatial derivative at each voxel, for each timepoint. This is done separately along each of (X,Y,Z) axes, generating three 4D datasets; each approximates signal change caused by head motion < 1 voxel along one axis, for every brain volume. PCA is performed on each timeseries, and the first eigenimage retained, which typically accounts for >95% of total variance. This produces 3 derivative eigenimages, stored in matrix ***G*** (*N* voxels x 3).
   2. Measure correlation with edge artifact: Perform spatial Canonical Correlations Analysis (CCA) between the optimal SPM (with MC) and ***G***. This is the multivariate extension of Pearson correlation, which gives canonical correlation ρ_k, mc_ between the SPM and the linear combination of ***G*** vectors that is most correlated with the SPM (which best approximates any motion artifact present in the SPM).

Given *K* artifact-correlation values with MC applied {ρ_k, mc_}, remove any outliers greater than 3 standard deviations above the mean; this accounts for cases where MC entirely fails to control motion (at *p*=0.001), assuming this is relatively rare. Then, record the highest remaining value in {ρ_k, mc_} for the current dataset. This value, denoted **ρ_max_**, is our threshold for significant motion artifact.

1. Identify any subject that is optimized without MC. Estimate {ρ_k, nomc_} as outlined in (**2**) for each subject without MC. If ρ_nomc_ < **ρ_max_**, keep this new pipeline; otherwise, select a new optimum out of all possible pipelines for this subject with ρ < **ρ_max_**. Perform the same testing and re-optimization for subjects with MC identified as outliers in (**2**).

This model provides a conservative test for TCM artifact that may be adapted to any dataset or pipeline; for current results, we corrected 2/24 (*strong* contrast) and 13/24 (*weak* contrast) subjects.
